# Prediction of Muscle Fatigue during Minimally Invasive Surgery Using Recurrence Quantification Analysis

**DOI:** 10.1155/2016/5624630

**Published:** 2016-05-24

**Authors:** Ali Keshavarz Panahi, Sohyung Cho

**Affiliations:** Industrial Engineering, Southern Illinois University Edwardsville, Edwardsville, IL 62026, USA

## Abstract

Due to its inherent complexity such as limited work volume and degree of freedom, minimally invasive surgery (MIS) is ergonomically challenging to surgeons compared to traditional open surgery. Specifically, MIS can expose performing surgeons to excessive ergonomic risks including muscle fatigue that may lead to critical errors in surgical procedures. Therefore, detecting the vulnerable muscles and time-to-fatigue during MIS is of great importance in order to prevent these errors. The main goal of this study is to propose and test a novel measure that can be efficiently used to detect muscle fatigue. In this study, surface electromyography was used to record muscle activations of five subjects while they performed fifteen various laparoscopic operations. The muscle activation data was then reconstructed using recurrence quantification analysis (RQA) to detect possible signs of muscle fatigue on eight muscle groups (bicep, triceps, deltoid, and trapezius). The results showed that RQA detects the fatigue sign on bilateral trapezius at 47.5 minutes (average) and bilateral deltoid at 57.5 minutes after the start of operations. No sign of fatigue was detected for bicep and triceps muscles of any subject. According to the results, the proposed novel measure can be efficiently used to detect muscle fatigue and eventually improve the quality of MIS procedures with reducing errors that may result from overlooked muscle fatigue.

## 1. Introduction

Since minimally invasive surgery (MIS) is performed using an endoscope and several thin instruments through small incisions made in patients, it provides patients with some advantages like shorter recovery time, less postoperative pain, earlier resumption of normal activity, and cost savings. However, it is more ergonomically challenging to performing surgeons due to its inherent complexity such as limited work volume and degree of freedom. Specifically, such complexity induces abnormal movements of arm and shoulder while holding certain body postures, for example, head and torso, for prolonged time [1–3]. Importantly, this complexity often exposes performing surgeons to increased ergonomic risks including muscle fatigue that can result in critical errors during surgical procedures [4–6]. The vulnerability to ergonomic risk is well confirmed in a literature stating that “performing laparoscopic surgery is significantly more stressful for the surgeon than open surgery” [7]. In addition, it has been reported that MIS carries more complications than open surgery [8, 9]. Therefore, it is important to quantitatively measure ergonomic risks on performing surgeons, particularly muscle fatigue during MIS procedures. Note that timely intervention with information about muscle fatigue can ensure improved quality of MIS procedures.

**Figure 1:**
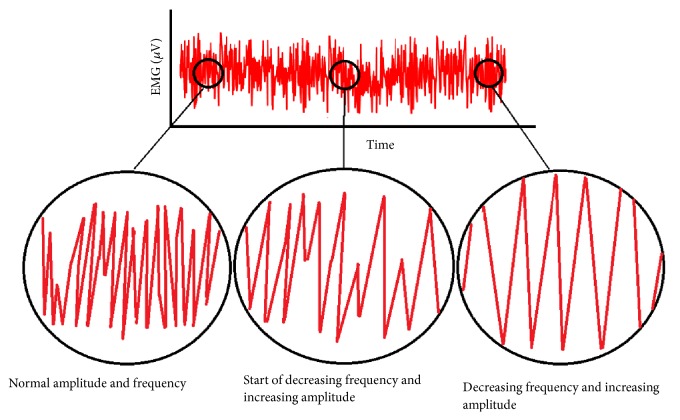
Schematic of changes in EMG data during muscle fatigue.

Some existing study for fatigue analysis has focused on quantitative measures and their applications for isometric or isotonic contractions during relatively short bouts of high-force activities. Among these studies, frequency banding analysis predicted muscle fatigue when lower frequency bands increase and higher frequency bands decrease [10]. Standard discomfort analysis was also used to detect the level of muscle fatigue on subjects, which may lack objective analysis [11]. In addition, heart rate [12] and tissue oxygen saturation [13] were used as indicators of fatigue development. Importantly, power spectral density (PSD) of transformed EMG data through Fast Fourier Transform approach was widely used to detect muscle fatigue [14–16]. In this approach, frequency and amplitude changes are considered as a result of a reduction in conduction velocity in the muscle fibers and larger motor unit synchronization.  Figure 1 schematically depicts these changes in various stages of the muscle fatigue.

As MIS operations can be considered as prolonged light muscle activation, PSD based measure, which is mainly for static activities, may not be efficiently used to predict muscle fatigue during MIS operations. Specifically, when PSD based measures are used for light muscle activations, conflicting results and complicated relationship between subjective and objective fatigue have been reported [17, 18]. Such conflicts result from stationary data requirement for PSD based measures to be successfully used. In fact, EMG data collected from light muscle activations are nonstationary due to changing distance between muscle and EMG sensor, as well as changing muscle length. From the literature, it is noted that fatigue mechanism during these activities is not well understood in comparison to high-force (or intense) muscle activities [19]. Although there are no significant reductions in muscle force level during MIS operations, there are some physiological changes in muscles that can lead to muscle fatigue affecting the capacity of muscles and the performance of subjects [20, 21]. Uhrich et al. [22] assessed the muscle fatigue during a relatively short time during simulated laparoscopic surgery. In their study, the effects of fatigue, monitor placement, and surgical experience have been compared. After obtaining the EMG activity and muscular discomfort scores before and after a fatigue session, it was found that the EMG data and discomfort scores demonstrated a fatigue response in several muscle groups. They found minimal differences between the two monitor positions and less muscle activity and discomfort in the attending surgeons. In another study, Slack et al. [23] studied the effect of operating time on surgeon's muscular fatigue. The length of the operations varied within 1–10 hr, but only one minute period before and after operations were analyzed using frequency analysis. They found that brachioradialis was being used more and fatigued faster. In addition, the muscle fatigue increases proportional to time. On the other hand, recurrence quantification analysis (RQA) has been tested on biceps brachii and shown to be more sensitive to muscle fatigue than FFT variable spectral center frequency (fC) [24]. In another study, Filligoi and Felici [25] showed that Determinism%, which is one of the RQA variables, is more effective than median frequency to detect EMG signal changes in biceps brachii muscle. For the first time, this novel data analysis method is used in our study to quantify any possible muscle fatigue in real laparoscopic surgery operations which is known as prolonged and low-force muscle activity compared to some intense tasks which involve isometric muscle contraction throughout the whole task.

The main goal of this study is to answer the following questions:

- Can objective manifestations of muscle fatigue be detected from EMG data during a laparoscopic surgery as prolonged light muscle activation?
- What is time-to-fatigue for the muscles experiencing fatigue during laparoscopic surgery?
- Which muscle has the highest possibility level of fatigue in laparoscopic surgery among the tested muscle groups?

## 2. Methods

As the first step, EMG data for fatigue analysis was collected. Specifically, surface EMG electrodes were attached to upper arm muscles of participants to collect muscle activations while performing various MIS procedures. Next, EMG data was converted into higher dimensional data using time shift and represented by recurrence plots that facilitate recurrence quantification analysis (RQA), particularly computation of determinism values. Then, moving average technique was applied for trajectories of determinism values to detect any changes as the indicator of muscle fatigue.

### 2.1. EMG Data Collection

Under an IRB-approved protocol, five right-hand-dominant expert laparoscopic surgeons, who have performed more than 100 laparoscopic surgeries, performed fifteen MIS procedures in a local hospital.  Table 1 shows more details about those surgical operations.

On each participant, a total of eight surface EMG (sEMG) electrodes were attached to the following four bilateral muscles including bicep, triceps, deltoid, and trapezius. Note that since lower arms are scrubbed from the fingertips to the elbow, electrodes were not placed on these sterilized muscle compartments. Tricep and bicep were selected due to their high activity level during arm flexion and extension. Since the shoulder and neck are the common area of muscle fatigue, trapezius and deltoid were also included in our study. All sEMG data were collected using an 8-channel Bioradio® 150 physiologic data acquisition system (Great Lakes Neurotechnologies, Incorporated®, Cleveland, OH). After wiping skin surfaces overlying target muscle groups with rubbing alcohol and allowing the alcohol to dry, two 1′′ × 1′′ MVAP-II® electrodes (MVAP Medical Supplies, Incorporated, Newbury Park, CA) were placed over the muscle bellies of each muscle group and connected to the positive and negative input poles for each channel. An electrode was also attached to the right elbow and connected to the ground input on the Bioradio 150 to complete the input circuit. The Biocapture® data acquisition software package (Great Lakes Neurotechnologies, Incorporated, Cleveland, OH) was used to capture and filter sEMG data. More specifically, sEMG data was sampled at frequency of 256 Hz from each channel. Digital signal processing filters were then applied to exclude the noise that are at low (<10 Hz) and high frequency (>127 Hz) signals.

### 2.2. Recurrence Quantification Analysis (RQA)

**Figure 2:**
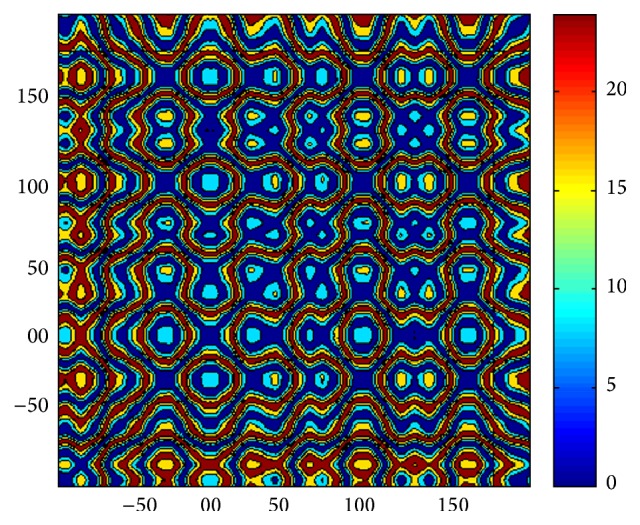
Example of a recurrence plot.

RQA is a time series analysis method and detects the deterministic structure of the dynamical systems. It also provides some valuable information from recurrence plots [26]. The principle of recurrence plots analysis can be summarized as follows. Let *x*(*i*) be the *i*th point on the orbit describing a dynamical system in *d*-dimensional space, for *i* = 1,…, *N*. The recurrence plot is an array of dots in a *N* × *N* square, where a dot is placed at (*i*, *j*) whenever *x*(*j*) is sufficiently close to *x*(*i*). In order to obtain a recurrence plot from a time series {*u*_*i*_}, the following procedures are required. First, we choose an embedding dimension *d* and construct the *d*-dimensional orbit of *X*(*i*) by the method of time delays: if *u* and *i* are scalar, *x*_(*i*)_ = (*u*_*i*_, *u*_*i*+1_,…, *u*_*i*+*d*−1_). Next, we choose *r*_(*i*)_ such that the ball of radius *r*_(*i*)_ centered at *x*_(*i*)_ in *R* ^*d*^ contains a reasonable number of other points *x*_(*j*)_ of the orbit. Finally, we plot a dot at each point (*i*, *j*) for which *x*_(*j*)_ is in the ball of radius *r*_(*i*)_ centered at *x*_(*i*)_. We call this picture a recurrence plot (RP) and an example of RPs is shown in Figure 2. Note that *i* and *j* are, in fact, times; therefore, a recurrence plot describes natural time correlation information.

Recurrence plots tend to be fairly symmetric with respect to the diagonal *i* = *j* because if *x*_(*i*)_ is close to *x*_(*j*)_, then *x*_(*j*)_ is close to *x*_(*i*)_. There is, however, no complete symmetry because we do not require *r*_(*i*)_ = *r*_(*j*)_ [27]. When *N* is more than 2 dimensions in a phase space, projection is performed. Starting from the time series *s*_(*t*)_ = {*s*_1_,…, *s*_*n*_}, the attractor of the underlying dynamics is reconstructed in a phase space by applying the time-delay vector method by Takens [28]. The reconstructed trajectory *X* can be expressed as a matrix where each row is a phase space vector:

(1) $$\begin{array}{c} X={\left[\;x_{1},x_{2},\ldots x_{m}\;\right]}^{T}, \end{array}$$

where *x*_*i*_ = [*s*_1_, *s*_*i*+*T*_,…*s*_*i*+(*D*_E_ − 1)*T*_]^*T*^, *m* = *n* − (*D*_E_ − 1)*T*, *D*_E_ is the embedding dimension, and *T* is the delay time. The recurrence plot is a tool that can be used to investigate higher dimensional dynamics through a two-dimensional binary plot of its recurrences. Any recurrence of state *i* with state *j* is pictured on a Boolean matrix expressed by

(2) $$\begin{array}{c} R_{i,j}^{D_{\text{E}},\varepsilon }=\Theta \left(\;\varepsilon -\left\|\;x_{i}-x_{j}\;\right\|\;\right), \end{array}$$

where *x*_*i*,*j*_ ∈ *R* ^*D*_E_^ are the embedded vectors, *i*, *j* ∈ *N*, Θ(·) is the Heaviside step function, and *ε* is an arbitrary threshold. In the graphical representation, each nonzero entry of *R*_*i*,*j*_ is marked by a black dot in the position *i*, *j*. Since any state is recurrent with itself, the recurrent plot (RP) matrix fulfills *R*_*i*,*j*_ = 1 which hence contains the diagonal line of identity (LOI).

**Figure 3:**
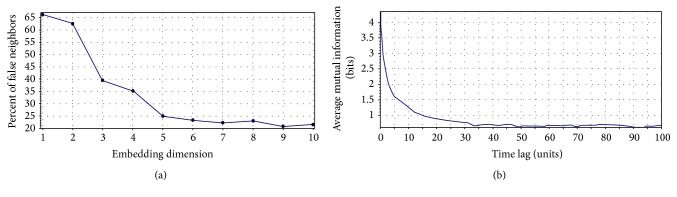
Optimal embedding dimension using FNN (a) and time delay using AMI (b).

The percentage of determinism (%DET) derived from diagonal lines and related to predictability of the system is an important parameter in RQA which quantifies the ratio of the recurrence points. The following formula is typically used to calculate %DET from a recurrence plot [26]:

(3) $$\begin{array}{c} \%\text{DET}=\frac{\sum_{l=l_{min}}^{N}lP\left(l\right)}{\sum_{l=1}^{N}lP\left(l\right)}\times 100\left(\;\%\;\right), \end{array}$$

where *P*(*l*) is histogram or frequency distribution of diagonal line lengths, *N* is the length of a data series, and *l*_min⁡_ is predefined minimal length of a diagonal line. In this study, the above %DET for EMG data was used as a muscle fatigue indicator. Cross recurrence plot (CRP) toolbox available in MATLAB was used for RQA analysis [26]. In order to ensure optimized results, RQA parameters including embedding dimension (*D*_E_), time delay (*T*), and threshold (*ε*) should be selected carefully. In the CRP toolbox, one can set the parameters of optimal embedding dimension and time delay to the obtained values from false nearest neighbors (FNN) and average mutual information (AMI) function which may lead to the optimal recurrence plots as shown in Figure 3.

Using these functions, embedding dimension and time-delay parameters were set to 10 and 4, respectively. To facilitate optimal threshold value, it has been suggested to consider the threshold value only a few percent of the maximum phase space diameter [29]. Therefore, the threshold value was chosen according to the 10% of the minimum value of maximum phase space diameter of the data. In this study, the minimum number of points constructing a deterministic diagonal line was set to two.

The whole duration of MIS operations summarized in Table 1 was analyzed by applying RQA for each minute of the operation with a window length of 15,360 data points. Then, %DET value was derived from RQA analysis and the results were plotted against operation time. Finally, a moving average analysis with the interval (or window size) of ten was applied to the %DET values to determine time-to-fatigue.

## 3. Results

**Figure 4:**
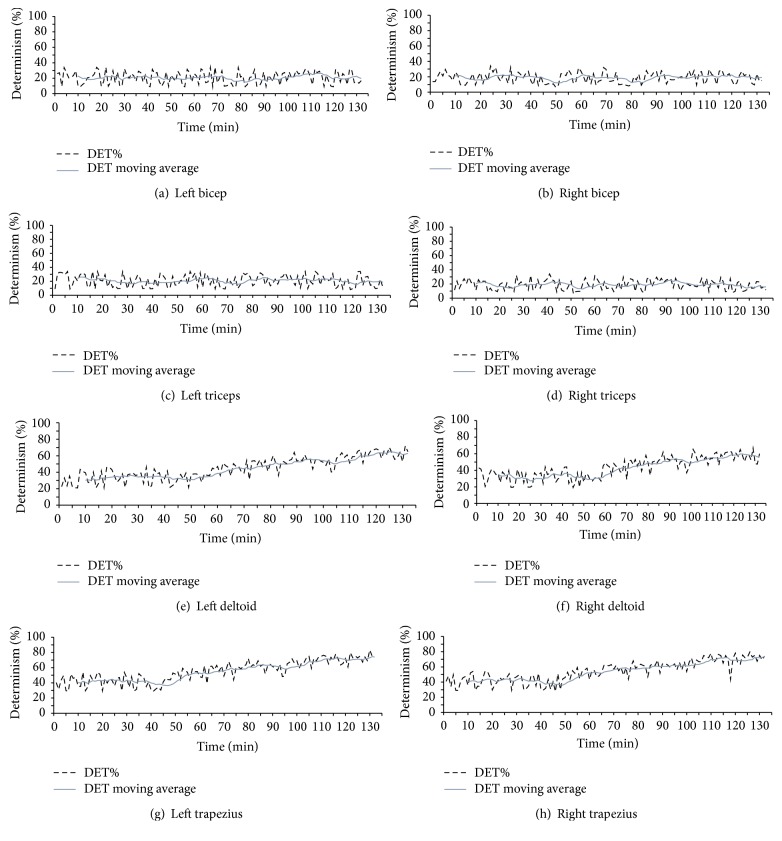
%DET value for all the muscle groups of surgeon 1, case 3. The vertical axis is Determinism % and the horizontal axis is time (min). (a) left bicep, (b) right bicep, (c) left triceps, (d) right triceps, (e) left deltoid, (f) right deltoid, (g) left trapezius, and (h) right trapezius.

Figure 4 shows %DET values of all eight muscles for surgeon 1 performing case  3 for 132 minutes as an example. It was observed from Figures 4(g) and 4(h) that moving average of %DET of bilateral trapezius muscles increased after 45–50 minutes of operation. In addition, moving average of %DET of bilateral deltoid increased after 55–60 minutes of operation as shown in Figures 4(e) and 4(f). Between deltoid and trapezius, bilateral trapezius became more deterministic with %DET close to 75% at the end of operation while bilateral deltoid was less deterministic with %DET value close to 50%. In this study, the increase in the moving average of %DET values is considered as the development of muscle fatigue. Thus, it is conjectured from these observations that trapezius became more fatigued than deltoid for surgeon 1 performing case  3. However, there were no changes in moving averages of %DET of bilateral bicep and triceps throughout the entire operation as shown in Figure 4.

**Figure 5:**
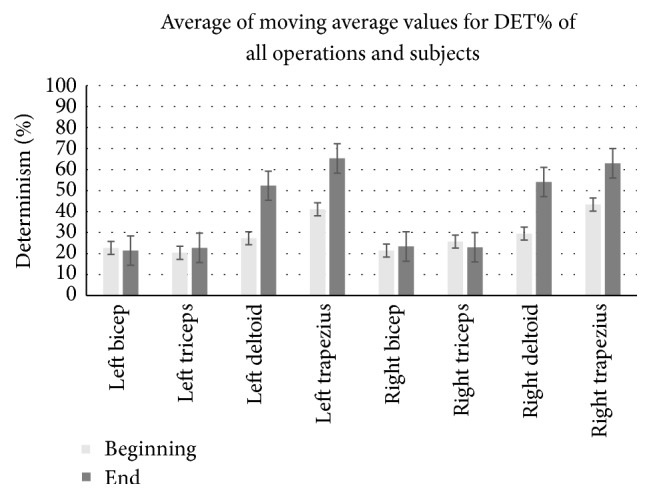
Average of moving average values for DET% of all the operations and subjects for the beginning and end of operations.

As the next step, %DET was tested for all the MIS operations and results are summarized in Table 2. DET results did not detect any changes in any of the muscles for surgeon 2 in case number 1 which had the least completion time of 55 minutes. Also, surgeon 4 in case number 2 as well as surgeon 5 in case number 3 did not experience any fatigue in their deltoid muscle. Specifically, no fatigue signs were detected on bilateral bicep and triceps in any of the subjects.  Figure 5 shows the mean of moving average values for DET% of all the operations and subjects. Table 2 also shows that fatigue signs are developed at least 45 minutes after operations begin and trapezius gets fatigue sign earlier than deltoid.

## 4. Discussion

The moving average of %DET for bilateral trapezius deltoid increased after 45–50 and 55–60 minutes of operation, respectively. This might be caused due to the following nature of MIS procedures: (i) during MIS, surgeons should maintain their head positions at certain orientation to keep watching the monitor and this may impose significant stress on trapezius muscle, and (ii) as the surgeon operates thin and long instrument through a small incision, they often use excessive shoulder movements to overcome the limited degree of freedom and this may impose fatigue on deltoid muscle. In this study, higher %DET value implies that the EMG data are getting more deterministic and periodic. This deterministic and periodic pattern is the result of synchronization of motor units in fatiguing stage [30]. In order to supply necessary force to continue certain tasks, more motor units are involved during muscle fatigue. If almost all motor units have already been involved, motor unit synchronization takes place to continue task operation and prevent the failure. The fact that more motor units are recruited and synchronized during the course of muscle fatigue has been shown in literature [31]. For conventional occupational tasks, increase in synchronization of motor units has been successfully detected using %DET values through a computer simulation model [32].

On the other hand, shoulder disorder is considered as one of most important musculoskeletal disorders, especially in prolonged repetitive activations with high precision [33–35]. An increase in interstitial potassium concentrations in trapezius muscle can be the cause of decreased conduction velocity [36]. Also, the intra- and extracellular sodium and potassium concentration changes can be the main reason for changes in EMG data which are related to muscle fatigue [37]. Although the recovery of these metabolic changes is quick, in prolonged light muscle activation, muscles need longer recovery times in order to regain the primary force capacity [37, 38]. Because of orderly recruitment of motor units, low threshold fibers are vulnerable in prolonged muscle activation, which necessitates the importance of recovery time to avoid myalgic disorders in trapezius [39, 40]. It can be summarized from the literature review that fatigue analysis is highly difficult for prolonged light muscle activation tasks such as MIS procedures.

This study shows that trapezius was the first muscle to show fatigue sign in prolonged light muscle activation during MIS procedures. It is interesting to note that similar results were reported in a light assembly task in industrial operations [41]. While the existing study also suggested recovery/break time for workers at 90 minutes after the task begins, the results in this study suggest recovery/break time after 45–50 minutes after MIS operations begin. More likely, this difference results from intensity and precision level of two tasks: industrial and surgical. Bicep and triceps did not experience any muscle fatigue. This might be because of less muscle activation and less muscle contraction in laparoscopic surgery for these two muscles.

As muscles are vulnerable to fatigue during MIS operations which have prolonged and intense nature, detecting the muscle fatigue and estimation of time-to-fatigue are of great importance. To meet this need, this study proposed and tested a novel measure that can be efficiently used for detection of muscle fatigue and time-to-fatigue from EMG data. In the future study, correlation between objective and subjective results would be important. Here, objective results mean fatigue analysis using the proposed measure of this study and subjective results correspond to survey analysis to subjects asking whether they feel fatigue symptoms during MIS procedures. Also, it would be interesting to compare performance analysis with fatigue analysis in the future, since the final goal for muscle fatigue analysis is to detect any possible effect on surgeon's performance. This will emphasize the importance of quantitative muscle fatigue analysis, although it might be challenging to apply performance analysis to real surgical operations. However, performance analysis can be easily applied to dry lab experiments such as Fundamentals of Laparoscopic Surgery (FLS) tasks, since there are validated methods to analyze the performance of subjects. Therefore, the combination of RQA, as a quantitative muscle fatigue analysis method, and standard performance analysis of FLS tasks will open up new doors for future studies related to muscle fatigue and performance analysis of real MIS operations which would be very beneficial for surgical community. Also, future studies should include larger number of subjects and more surgical operations in order to confirm findings of this study.

## 5. Conclusion

In this study, recurrence quantification analysis (RQA) was applied to EMG data recorded from eight muscle groups of five surgeons while doing fifteen MIS operations. The results showed that this novel measure could detect the sign of muscle fatigue on bilateral deltoid and trapezius at 45–55 minutes after operations began, and no sign of fatigue was found on other muscles. It was found from this study that trapezius and deltoid were the most vulnerable muscles among all eight muscle groups tested. Here, it is worthwhile to note the nature of MIS procedures such that surgeons manipulate long and thin instruments for the frequent changes of their orientation and position mainly using bicep and triceps and maintain their arm posture using deltoid while gazing through monitor using trapezius muscle for prolonged time. Considering this nature, deltoid and trapezius may show clear sign of fatigue while other muscles do not show any sign of fatigue as tested in this study. Based on the results, it could be suggested that surgeons need to take break time at 45–50 minutes after MIS operations begin in order to minimize muscle fatigue and other possible muscle disorders. To have a better understanding about the effect of recovery time on surgeons doing such a complicated laparoscopic operation, future research might test different recovery times to find an optimum amount of time for muscle relaxation before muscles fatigue. In addition, ergonomic improvement of surgical equipment to reduce the intensity level might be efficient in reducing muscle fatigue or at least increasing time-to-fatigue.

## Figures and Tables

**Table 1:** MIS operations from which EMG data was collected^*∗*^.

Subject	Lap #	Completion time (min)	Description
Surgeon 1	Lap 1	96	Ventral hernia repair
	Lap 2	113	Cholecystectomy
	Lap 3	132	Inguinal hernia repair

Surgeon 2	Lap 1	55	Sleeve gastrectomy
	Lap 2	66	Sleeve gastrectomy
	Lap 3	128	Sleeve gastrectomy

Surgeon 3	Lap 1	131	Heller myotomy, Dor fundoplication, and liver biopsy
	Lap 2	152	Ventral hernia repair
	Lap 3	171	Paraesophageal hernia repair

Surgeon 4	Lap 1	122	Sleeve gastrectomy
	Lap 2	101	Inguinal hernia repair
	Lap 3	88	Sleeve gastrectomy

Surgeon 5	Lap 1	71	Heller myotomy
	Lap 2	116	Ventral hernia repair
	Lap 3	144	Cholecystectomy

^*∗*^Ventral hernia repair: the hernia is repaired by mesh or sutures entered through instruments placed into small incisions in the abdomen. Cholecystectomy: to remove the gallbladder using several small incisions. Inguinal hernia repair: to repair a hernia in the abdominal wall of the groin. Sleeve gastrectomy: to remove a large portion of the stomach to help with weight loss. Heller myotomy: to treat the achalasia by cutting the muscles of the cardia to allow food and liquid to pass to the stomach. Dor fundoplication: to prevent reflux from the stomach into the esophagus by partially wrapping the stomach around the esophagus. Paraesophageal hernia repair: the diaphragm at the esophageal hiatus is closed to prevent the stomach from reherniating, and then the fundoplication is performed to keep the stomach from herniating back into the chest cavity.

**Table 2:** Time-to-fatigue for all the muscle groups (minutes).

Surgeon	Case	L-bicep	R-bicep	L-tricep	R-tricep	L-deltoid	R-deltoid	L-trap	R-trap
1	1	N/F	N/F	N/F	N/F	*F*(55–60)	*F*(55–60)	*F*(45–50)	*F*(45–50)
	2	N/F	N/F	N/F	N/F	*F*(55–60)	*F*(55–60)	*F*(45–50)	*F*(45–50)
	3	N/F	N/F	N/F	N/F	*F*(55–60)	*F*(55–60)	*F*(45–50)	*F*(45–50)

2	1	N/F	N/F	N/F	N/F	N/F	N/F	N/F	N/F
	2	N/F	N/F	N/F	N/F	*F*(55–60)	*F*(55–60)	*F*(45–50)	*F*(45–50)
	3	N/F	N/F	N/F	N/F	*F*(55–60)	*F*(55–60)	*F*(45–50)	*F*(45–50)

3	1	N/F	N/F	N/F	N/F	*F*(55–60)	*F*(55–60)	*F*(45–50)	*F*(45–50)
	2	N/F	N/F	N/F	N/F	*F*(55–60)	*F*(55–60)	*F*(45–50)	*F*(45–50)
	3	N/F	N/F	N/F	N/F	*F*(55–60)	*F*(55–60)	*F*(45–50)	*F*(45–50)

4	1	N/F	N/F	N/F	N/F	*F*(55–60)	*F*(55–60)	*F*(45–50)	*F*(45–50)
	2	N/F	N/F	N/F	N/F	N/F	N/F	*F*(45–50)	*F*(45–50)
	3	N/F	N/F	N/F	N/F	*F*(55–60)	*F*(55–60)	*F*(55–60)	*F*(55–60)

5	1	N/F	N/F	N/F	N/F	*F*(55–60)	*F*(55–60)	*F*(55–60)	*F*(55–60)
	2	N/F	N/F	N/F	N/F	*F*(55–60)	*F*(55–60)	*F*(55–60)	*F*(55–60)
	3	N/F	N/F	N/F	N/F	N/F	N/F	*F*(55–60)	*F*(55–60)

Note: N/F: no fatigue.
